# Analysis of measured high-resolution doublet rovibronic spectra and related line lists of ^12^CH and ^16^OH†

**DOI:** 10.1039/d2cp02240k

**Published:** 2022-07-25

**Authors:** Tibor Furtenbacher, Samuel T. Hegedus, Jonathan Tennyson, Attila G. Császár

**Affiliations:** MTA-ELTE Complex Chemical Systems Research Group Pázmány Péter sétány 1/A H-1117 Budapest Hungary attila.csaszar@ttk.elte.hu; Department of Physics and Astronomy, University College London Gower Street London WC1E 6BT UK j.tennyson@ucl.ac.uk; Laboratory of Molecular Structure and Dynamics, Institute of Chemistry, ELTE Eötvös Loránd University and MTA-ELTE Complex Chemical Systems Research Group Pázmány Péter sétány 1/A H-1117 Budapest Hungary

## Abstract

Detailed understanding of the energy-level structure of the quantum states as well as of the rovibronic spectra of the ethylidyne (CH) and the hydroxyl (OH) radicals is mandatory for a multitude of modelling efforts within multiple chemical, combustion, astrophysical, and atmospheric environments. Accurate empirical rovibronic energy levels, with associated uncertainties, are reported for the low-lying doublet electronic states of ^12^CH and ^16^OH, using the Measured Active Rotational-Vibrational Energy Levels (Marvel) algorithm. For ^12^CH, a total of 1521 empirical energy levels are determined in the primary spectroscopic network (SN) of the radical, corresponding to the following seven electronic states: X ^2^Π, A ^2^Δ, B ^2^Σ^−^, C^2^ Σ^+^, D ^2^Π, E ^2^Σ^+^, and F ^2^Σ^+^. The energy levels are derived from 6348 experimentally measured and validated transitions, collected from 29 sources. For ^16^OH, the lowest four doublet electronic states, X ^2^Π, A ^2^Σ^+^, B ^2^Σ^+^, and C ^2^Σ^+^, are considered, and a careful analysis and validation of 15 938 rovibronic transitions, collected from 45 sources, results in 1624 empirical rovibronic energy levels. The large set of spectroscopic data presented should facilitate the refinement of line lists for the ^12^CH and ^16^OH radicals. For both molecules hyperfine-resolved experimental transitions have also been considered, forming SNs independent from the primary SNs.

## Abstract 2

Understanding the energy-levels and rovibronic spectra of the ethylidyne (CH) and the hydroxyl (OH) radicals is mandatory for a multitude of modelling efforts within multiple chemical, combustion, astrophysical, and atmospheric environments.

## Graphical abstract

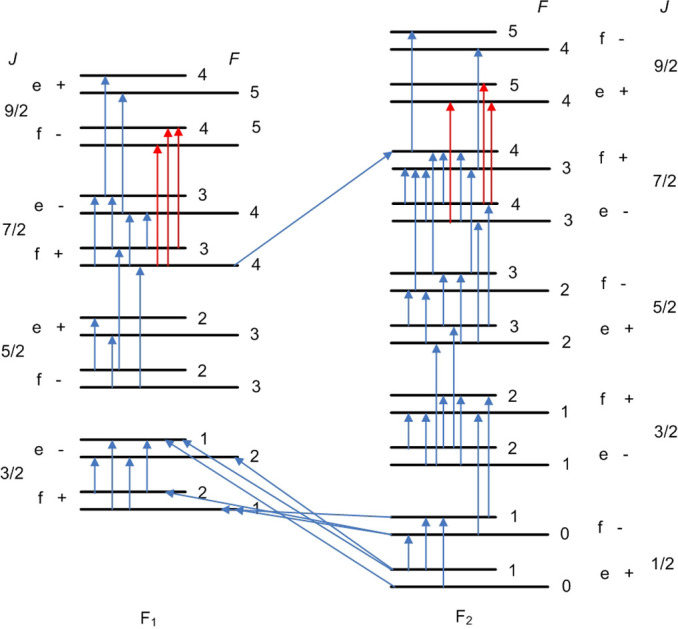

## 1 Introduction

The free radicals methylidyne (CH) and hydroxyl (OH) play central roles in multiple chemical, combustion, astronomical, and atmospheric environments exhibiting a wide range of thermochemical properties. Therefore, their rovibronic spectra have been studied in considerable detail by methods of high-resolution molecular spectroscopy. Here we collect and analyze these data using the Measured Active Rotational-Vibrational Energy Levels (Marvel) procedure.^1–5^

The CH radical has played a fundamental role in the furtherance of our scientific understanding during the last century. The radical's spectrum was identified in 1918.^6^ In 1937,^7^ methylidyne became the first molecule detected in interstellar space, prior to the proliferation of radio spectroscopy in the post-WWII period. The assignment of the broad 429.5–431.5 nm band, the so-called G-band of Fraunhofer, containing transitions corresponding to ^12^CH, was made 26 years before ^16^OH was detected using radio astronomy.^8^ Beyond its original discovery in the interstellar medium, CH has been detected in stellar atmospheres,^9^ including the Sun,^10,11^ in comets,^12^ in protostellar accretion disks,^13^ in planetary nebula,^14^ and in extragalactic sources.^15^ CH is used as a tracer for molecular hydrogen in the interstellar medium due to its origin being predominantly from the radiative association and recombination of C^+^ and H.^16^ It is also used as part of the classification of carbon giants.^17,18^ CH is important in further chemical and physical environments. For example, during combustion of hydrocarbons it is an important intermediate giving the flame its characteristic blue color.^19^ The ubiquity of CH across multiple environments, with a broad range of thermodynamic conditions, is why CH is one of the spectroscopically most studied diatomic molecules. Furthermore, this is why the proposed analysis of the rovibronic spectra of CH is so pertinent and broadly beneficial for future studies, both in helping to interpret observations and to improve astronomical, chemical, and physical models. Note in this respect that several hyperfine transitions of ^12^CH have been measured with an accuracy of a few Hz, providing a convenient way to put limits on the possible variation of the fine-structure constant and the electron-proton mass ratio with respect to time and local densities.^20^

OH is arguably the most important free radical in the Earth's atmosphere,^21,22^ governing atmospheric chemistry during the day. The OH radical is responsible for airglow^23,24^ and it is, together with HO_2_, one of the most dominant oxidizing agents of organic molecules in the troposphere.^25^ The hydroxyl radical is highly relevant for chemists as it has a significant role in the reactions characterizing combustion systems and flames^26,27^ and even in heterogeneous catalysis.^28^ OH is also of significant astrophysical interest, since it can be found in comets,^29^ stellar atmospheres,^30^ including the solar photosphere^31^ and sunspots,^32^ interstellar clouds,^33^ exoplanets,^27^ and planetary atmospheres.^34–36^ Accordingly, a large number of transitions of OH have been detected, the assigned spectra extend from resolved Λ-doubling and hyperfine transitions^37^ in the microwave (MW) to rotation (the “pure rotation” and the “spin-flip” branches), vibration–rotation, and rovibronic transitions.

Both OH and CH have key transitions at THz frequencies which have been studied from space using observatories such as the Kuiper Airborne Observatory,^38^ the ISO (Infrared Space Observatory),^39^ and Herschel,^40–42^ provoking dedicated laboratory studies.^43^ More recently, similar studies have been performed with the airborne SOFIA (Stratospheric Observatory for Far-Infrared Astronomy) observatory.^44–47^ These studies often investigate hyperfine-resolved transitions.

Spectroscopic parameters and line lists have been published for both radicals. There are no CH data reported in the spectroscopic database and information system HITRAN,^48^ while ^16^OH is molecule #13 in there and HITRAN2020^49^ lists altogether 55 698 lines in the range of 0–43 408 cm^−1^ for ^16^OH. The GEISA-2020 database^50^ is based on effective Hamiltonian (EH) calculations and contains a large number of data for OH. The most recent line list^9^ among those available,^9,51^ created for ^12^CH and ^13^CH, covers rovibronic transitions X–X, A–X, B–X, and C–X. As for OH, the available CH line lists cover transitions within the ground state^52^ as well as A–X electronic transitions.^53^ The ground-state transitions form the so-called Meinel bands,^54^ used, for example, to estimate OH rotational temperatures. The line lists of ref. 9, 52 and 53 were created as part of the MoLLIST project.^55^ The Jet Propulsion Laboratory (JPL) catalog^56^ contains data for both CH and OH.

A motivation for performing Marvel analyses of high-resolution spectra is the construction of high-accuracy line lists. Given that first-principles theoretical procedures can predict transition intensities with an accuracy competitive with state-of-the-art experiments,^57,58^ use of empirical energy levels, such as those determined by the Marvel procedure, to determine transition frequencies provides a route to constructing line lists of experimental quality. This is a major current objective of the ExoMol project.^59^ Note that similar Marvel-based studies have already been performed on second-row hydride radicals, like^9^ BeH,^60^ a radical important for fusion studies, and ^14^NH.^61^

**Fig. 1:**
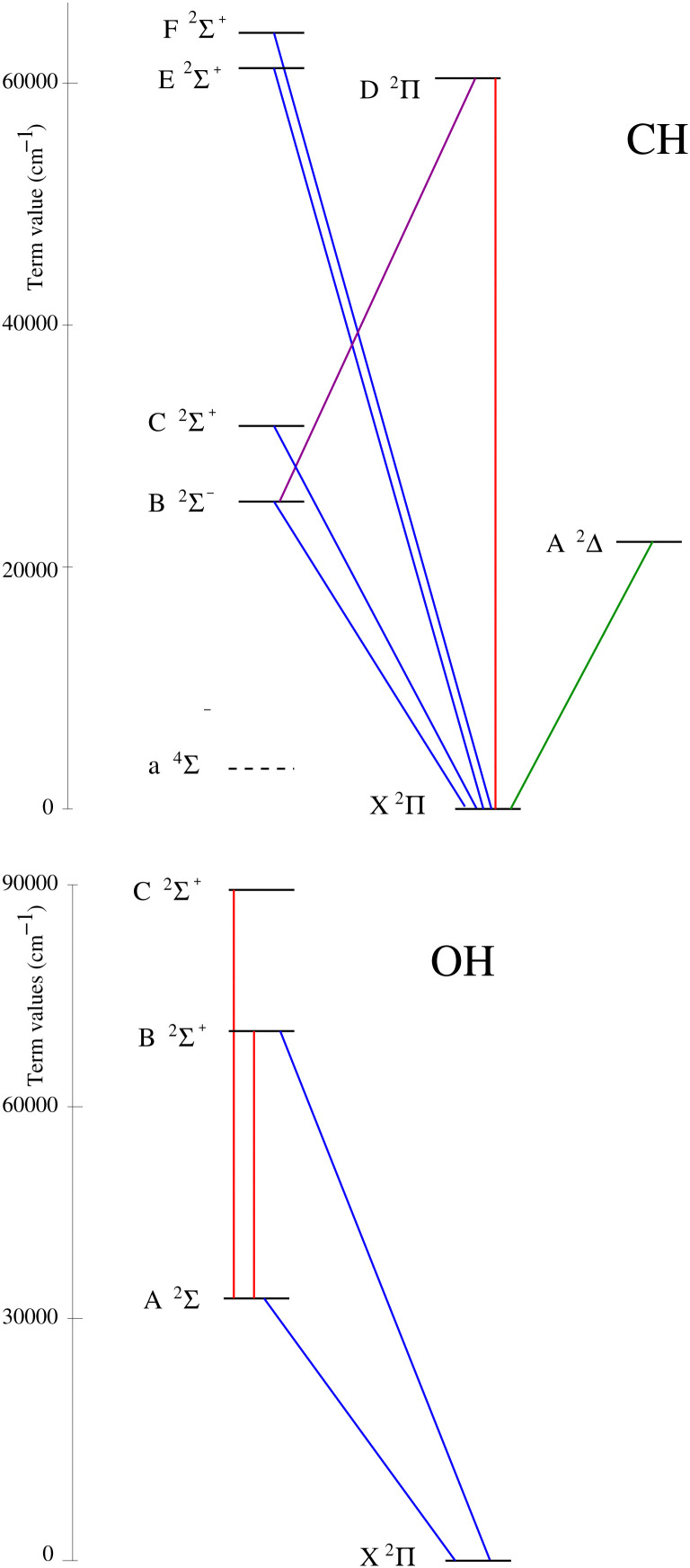
Schematic representation of rovibronic band systems for the radicals CH (top) and OH (bottom) considered in this study; the wavenumber scale of the term values is approximate. While high-quality spectroscopic data are available which involve the low-lying a ^4^Σ^−^ state of ^12^CH, there are no observed intercombination transitions connecting this state to the doublet states; therefore, these transitions are not treated here.

In this paper we undertake Marvel studies for the lowest seven doublet electronic states of CH and the lowest four doublet electronic states of OH (see Fig. 1 for the states covered in this study). To do this we analyze the experimental spectroscopic data available for the parent ^12^CH^6,9–15,20,40,51,62–105^ and ^16^OH^8,21,31,38,41,43–47,52,53,106–158^ isotopologues, using the Hamiltonian-free Marvel procedure,^1–5^ and obtain high-accuracy empirical rovibronic energy levels with well-defined uncertainties. The empirical energy levels obtained allow the critical assessment of line lists^9,51–53^ created previously for the two radicals. The principal aim of this study is to provide a set of validated experimental rovibronic transitions as well as a large number of empirical rovibronic energy levels, facilitating the construction of the next generation of line lists for ^12^CH and ^16^OH.

## 2 Methodological details

### 2.1 Marvel

The Marvel approach^1–5^ converts a set of assigned experimental transitions into empirical energy levels with associated uncertainties that are propagated from the input transitions to the output energy levels. This conversion relies on the construction of a spectroscopic network (SN),^3,159–161^ built upon the measured and assigned transitions.

### 2.2 Quantum numbers

Marvel requires that all transitions are assigned with a unique set of descriptors, usually quantum numbers, which are self-consistent across the entire dataset. A brief discussion of the labels we use is especially important as over time spectroscopists adopted different rules to assign the spectral lines of CH and OH, causing considerable confusion and making a global spectroscopic analysis error prone.

The electronic ground state of CH, X ^2^Π, has the following electron configuration: (1σ)^2^ (2σ)^2^ (3σ)^2^ (1π)^1^. The four lowest-energy electronic states of CH (see the top panel of Fig. 1), a ^4^Σ^−^ (*T*_e_ = 6024.40 cm^−1^), A ^2^Δ (*T*_e_ = 23148.7375 cm^−1^), B ^2^Σ^−^ (*T*_e_ = 24642.425 cm^−1^), and C ^2^Σ^+^ (*T*_e_ = 31809.6428 cm^−1^), all arise from a 1π ← 3σ excitation. In this list *T*_e_ is the electronic term and all *T*_e_ values are taken from ref. 9. During the present SN-based study, we only consider doublet states. There are three more doublet states of ^12^CH besides X, A, B, and C, all above 50 000 cm^−1^, which we considered: D ^2^Π, E ^2^Σ^+^, and F ^2^Σ^+^ (see the top panel of Fig. 1). In the literature, there has been some valid debate about the designation of the higher-lying Σ and Π electronic states of CH.^66,84,162,163^ While technically this may not be correct, we keep the traditional spectroscopic designation of the electronic states of CH, and, for example, denote the second ^2^Π state as D ^2^Π, with clear consequences for the designation of the higher ^2^Σ^+^ states.

It is most appropriate to treat the high-resolution spectra of ^12^CH as a diatomic having Hund's case (b) coupling.^37^ This means that *Ω* (= *Λ* + *Σ*) is not a good quantum number (*Λ* and *Σ* are the quantum numbers for the projection of the electronic orbital angular momentum **L** and the electron spin angular momentum **S** onto the internuclear axis, respectively). Furthermore, **L** is coupled to the overall rotational angular momentum **R** to form **N**, and **N** is then coupled to **S** to form the angular momentum **J** (**N** = **L** + **R** and **J** = **N** + **S**). Thus, to label the rovibronic states of ^12^CH within Marvel we use the following descriptors: the electronic state called *state*, *e.g.*, X2Pi, the vibrational quantum number (*v*), the total angular momentum quantum number (*J*), the rotationless parity (*e*/*f*),^164^ and values of F_1_ and F_2_ to label the spin components. We denote the F_1_ and F_2_ spin components by 1 and 2 in the Marvel transition file, respectively. Many CH spectra are assigned using the quantum number *N*, this can be used to give the spin components *J* = *N* + 1/2 and *J* = *N* − 1/2 corresponding to F_1_ and F_2_, respectively.^51^

The electronic ground state of OH, X ^2^Π, corresponds to the following electron configuration: (1σ)^2^ (2σ)^2^ (3σ)^2^ (1π)^3^. In this study, the rovibronic energy levels of the following four doublet states are considered: X ^2^Π, A ^2^Σ^+^, B ^2^Σ^+^, and C ^2^Σ^+^ (see the bottom panel of Fig. 1). The lower levels of ^16^OH are well represented by a Hund's case (a) coupling.^37^ The following descriptors were employed to label the rovibronic states of ^16^OH: state, *Ω*, *v*, *J*, and the rotationless parity^164^ (*e*/*f*). State designates the electronic state, *Ω* is the projection of the total angular momentum along the internuclear axis, and *J* is the total angular momentum quantum number without the nuclear spin. Regarding these descriptors we need to note the following: (1) in the case of ^16^OH, *Ω* can be either 1/2 or 3/2. The ground electronic state of ^16^OH is inverted (the spin-rotation constant of OH is negative); thus, the ^2^Π_3/2_ (F_1_) component lies below the ^2^Π_1/2_ (F_2_) component. (2) We follow the *e*/*f* scheme advocated in 78BrKaKeMi.^165^ This means that in the case of ^2^Π_1/2_ the order of parity (*e*/*f*) changes when *J* > 7/2. (3) In the case of ^2^Σ^+^ electronic states the *Ω* = 1/2 energy levels belong to the *f* parity (F_1_) and the *Ω* = 3/2 levels have *e* parity (F_2_).

## 3 Compilation of experimental sources

Our intention was to consider and analyse all literature sources of experimentally measured and assigned high-resolution ^12^CH and ^16^OH spectra. How far this was achieved is discussed separately for the two radicals.

### 3.1 ^12^CH

The structure of the CH band system treated in this study is displayed in Fig. 1 (see the top panel). The seven electronic states considered, X ^2^Π, A ^2^Δ, B ^2^Σ^−^, C ^2^Σ^+^, D ^2^Π, E ^2^Σ^+^, and F ^2^Σ^+^, are linked by a series of transitions, part of high-resolution, rotationally-resolved spectra. The lowest quartet state is the a ^4^Σ^−^ state, whose term value is only about *T*_e_ = 6024 cm^−1^.^9,163^ Nelis *et al.*^86^ recorded a far-infrared laser magnetic resonance spectrum containing 558 transitions within this state. Unfortunately, there are no reported intercombination transitions for CH linking the doublet and quartet manifolds. Thus, here we are concentrating on the SN formed by the seven doublet electronic states.

The full list of data sources employed in the final Marvel analysis^9,10,20,62,63,66,73,74,76–79,81,83,84,87,89–97,99,101,102,105^ of ^12^CH is given in Table 1, which also provides details of the range of wavenumbers and the number of transitions measured and validated for each vibronic band, with some statistical analysis of the uncertainties characterizing the source segments. After careful analysis, altogether 29 sources could be utilized, covering a total of 6348 transitions.

**Table 1:** Experimental sources, denoted with unique tags, used to construct the spectroscopic network of the hyperfine-unresolved rovibronic transitions of ^12^CH. Given are the wavenumber range (Range), in cm^−1^, of each source, the number actual (*A*) and validated (*V*) transitions, plus uncertainty (unc.) statistics, in cm^−1^, with Avg. = average and Max. = maximum

Tag^a^	Range	*A*/*V*	Avg. unc.	Max. unc.
13TrHeHiTa^102^	0.02–0.11	2/2	2.96 × 10^−9^	4.92 × 10^−9^
06McMoBrTh^99^	0.11–0.49	3/3	7.04 × 10^−7^	1.00 × 10^−6^
85StWoBr^79^	0.11–0.11	1/1	1.69 × 10^−4^	1.69 × 10^−4^
83BrBr^74^	0.16–0.81	4/4	2.01 × 10^−4^	5.98 × 10^−4^
84BrBr^76^	0.24–23529.83	82/81	5.59 × 10^−3^	2.80 × 10^−2^
83BoDeDe^73^	1.46–2.54	5/5	9.34 × 10^−6^	1.33 × 10^−5^
95Zachwiej_C^91^	14.27–15781.09	138/138	5.58 × 10^−3^	1.58 × 10^−2^
00Amano^96^	17.77–17.91	2/2	8.56 × 10^−5^	1.50 × 10^−4^
13TrHeToLe^20^	17.77–17.91	2/1	3.50 × 10^−7^	3.50 × 10^−7^
01DaEvBr^97^	67.07–141.78	8/8	5.78 × 10^−5^	1.33 × 10^−4^
10CoBe^101^	2093.93–3036.75	205/204	2.04 × 10^−2^	5.26 × 10^−2^
91BeBrOlHa^89^	2162.60–27561.99	572/570	9.84 × 10^−3^	7.36 × 10^−2^
87Bernath^83^	2309.84–2953.36	183/183	5.52 × 10^−3^	1.46 × 10^−2^
89MeGrSaFa^10^	2332.12–3037.34	378/378	5.38 × 10^−3^	3.50 × 10^−2^
84LuAm^77^	2580.65–2937.06	54/54	1.15 × 10^−3^	3.28 × 10^−3^
96KePaRyZa^92^	20202.55–27562.00	231/228	1.73 × 10^−2^	1.33 × 10^−1^
95Zachwiej^91^	20232.36–24007.21	1015/974	1.36 × 10^−2^	2.80 × 10^−1^
14MaPlVaCo^9^	21842.11–31628.30	679/535	5.11 × 10^−1^	2.04
41Gero^63^	22372.25–27561.60	1108/1091	1.29 × 10^−1^	1.59
90BeKePaRy^87^	23037.98–23878.85	557/498	6.14 × 10^−2^	4.58 × 10^−1^
98KuHsHuLe^94^	24475.90–27548.20	111/93	2.23 × 10^−1^	5.78 × 10^−1^
91Para^90^	25348.38–25823.34	59/50	8.45 × 10^−3^	2.28 × 10^−2^
99LiKuHsLe^95^	30980.42–32048.74	124/116	2.00 × 10^−1^	2.00 × 10^−1^
19MeLiUb^105^	31002.32–32269.09	213/212	6.85 × 10^−2^	5.00 × 10^−1^
32Heimer^62^	31049.00–32388.47	159/133	2.00 × 10^−1^	2.00 × 10^−1^
69HeJo^66^	31226.14–64621.60	150/115	3.78 × 10^−1^	4.14
97BeKeRy^93^	31387.80–32202.22	63/50	3.55 × 10^−2^	8.50 × 10^−2^
86UbMeTeDy^81^	31677.57–31908.06	35/32	3.17 × 10^−3^	8.25 × 10^−3^
85ChChCo^78^	63685.00–64155.00	48/46	4.568	8.87
87ChPaChCo^84^	63686.10–69110.30	157/149	2.04	7.99

a Tags denote experimental data-source segments employed during this study. The column ‘Range’ indicates the range (in cm^−1^) corresponding to validated wavenumber entries within the experimental transitions list. ‘*A*/*V*’ is an ordered pair, where *A* and *V* are the number of assigned and validated transitions related to a given source segment, respectively, obtained at the end of the Marvel analysis.

Some of the available sources had to be excluded from our final Marvel analysis. Table 2 lists these sources and gives a brief explanation for their exclusion. In a number of cases, like 65BlNi,^65^ the exclusion is due to the fact that the transitions reported are simply not available in the source. Other sources claimed to have measured highly-accurate and well-resolved transitions, but the accuracy of the transition data provided is not even close to the claimed uncertainty. Another case is the example of 88LyWo,^85^ who report successful measurement of the A ^2^Δ–X ^2^Π R1(5.5) transition with an uncertainty of 0.0002 cm^−1^ but with no actual transition wavenumber given.

**Table 2:** Details about the experimental sources on ^12^CH spectroscopy excluded during this study, with reason given for the exclusion

Source	Reason for exclusion
00Amano^96^	Criticised by 13TrHeHiTa^102^ as having large systematic errors.
08JaZiMcPe^100^	No extractable data; focuses on magnetic resonance.
14TrHeToHi^103^	Data provided by 13TrHeToLe.^20^
19MaRoBrMu^104^	Modelling/experimental study with no extractable data.
56KiBr^64^	Data are superceded with those from more accurate sources.
65BlNi^65^	No extractable data available from this source.
71BaBr^67^	Data are superceded with those from more accurate sources.
74RyElIrSu^69^	Astronomical measurement of lower accuracy than laboratory determinations.
78HoMuHeEv^70^	Data are superceded with those from more accurate sources.
83BrEv^75^	Only Hamiltonian parameters are provided.
83BrBr^74^	Calculated data only.
88LyWo^85^	No transitions data provided.
88NeBrEv^86^	Only Hamiltonian parameters are provided.
90NeBrEv^88^	Only Hamiltonian parameters are provided.
91GrLaSaVa^11^	No transitions data provided.

In addition to the observed sources provided in Table 1, additional ground-state transitions were incorporated into the final input transitions file, calculated from the effective Hamiltonian (EH) term values provided by Zachwieja.^91^ In the input transitions file, these lines are tagged as 95Zachwiej_C.*xx* and they are useful for two reasons. First, they help to fix the values of the ground-state energies, which is particularly important for ^12^CH, as it lacks a substantial list of MW transitions. An accurate set of ground-state energies helps determining whether a rovibronic transition is an outlier and therefore improves the self-consistency of the SN. Second, use of EH values allows two or more components of the SN to be unified, as they provide linking transitions that are otherwise not present among the observed ones. Utilization of EH energies does, however, have a drawback: we do not know the accuracy of the individual energy levels. In general, the energies of lower-*J* states are more accurately determined by the EH method than those of states with higher *J* values. When experimental results conflicted with an EH value, the effective Hamiltonian value was deleted. Our final list of transitions contains 138 transitions determined using the EH approach. It is the duty of future accurate measurements to replace as many of these artificial transitions as feasible.

### 3.2 ^16^OH

The full list of ^16^OH data sources used in the final Marvel analysis,^21,31,43,52,53,116,121,131,133,137–141,143–145,147,150,151,154^ along with some characteristics, is given in Table 3. Although we aim to process all experimentally measured transitions, sometimes the earlier experimental studies are just too inaccurate to justify their inclusion into the Marvel input transitions file. For example, in the case of the A^2^Σ^+^–X^2^Π band, the average uncertainty of the 9200 lines of 18YoBeHoMa^53^ is about 5 × 10^−4^ cm^−1^, while the accuracy of earlier works, for example, 34TaKo^166^ and 62DiCr,^167^ is significantly worse, only about 0.01−0.1 cm^−1^. Consequently, we decided to exclude these sources from our analysis. For similar reasons, we excluded the airglow^168–171^ and night-sky^23,54,172,173^ spectra of ^16^OH from our analysis, as well. Some papers recorded spectra but do not provide any line positions.^114,174^

**Table 3:** Experimental sources, denoted with unique tags, used to construct the spectroscopic network of hyperfine-unresolved rovibronic transitions of ^16^OH. Given are the wavenumber range (Range) of each source, the number actual (*A*) and validated (*V*) transitions, plus uncertainty (unc.) statistics, with Avg. = average and Max. = maximum, in cm^−1^

Source tag	Range/cm^−1^	*A*/*V*	Avg. unc.	Max. unc.
09BeCo_C^154^	0.06–36721.34	197/197	3.07 × 10^−3^	2.63 × 10^−2^
79KeCl^21^	51.40–147.85	41/41	9.45 × 10^−2^	2.10 × 10^−1^
11MaPiBaBr^43^	101.28–942.57	215/215	5.16 × 10^−4^	5.00 × 10^−3^
91HaWh^140^	101.30–330.30	28/28	2.96 × 10^−2^	7.00 × 10^−2^
95MeSaGrFa^145^	361.05–3407.62	620/620	3.61 × 10^−3^	6.20 × 10^−2^
97PoZoViTe^147^	396.57–563.87	19/19	5.00 × 10^−3^	5.00 × 10^−3^
09BeCo^154^	640.95–791.94	31/31	2.00 × 10^−3^	2.00 × 10^−3^
83GoMuLaDo^31^	814.32–961.66	31/31	8.55 × 10^−3^	1.05 × 10^−1^
85LeBoDe^133^	918.81–1095.03	38/38	2.66 × 10^−3^	3.05 × 10^−2^
94AbDaRaEn^143^	2066.66–8666.48	1925/1912	2.31 × 10^−3^	3.01 × 10^−1^
90AbDaRaEn^137^	2211.70–3922.00	295/286	5.00 × 10^−3^	5.11 × 10^−2^
76MaChMa^121^	2696.00–10358.28	1117/1057	1.05 × 10^−2^	1.13 × 10^−1^
84Amano^131^	3280.04–3767.76	38/38	5.55 × 10^−3^	1.50 × 10^−2^
01NiHaNe^150^	3558.07–3855.04	23/23	1.27 × 10^−2^	4.14 × 10^−2^
16BrBeWeSn^52^	4308.76–7154.83	351/351	8.00 × 10^−4^	2.61 × 10^−3^
02TeBeZoSh^151^	5540.67–6866.25	300/289	6.25 × 10^−3^	4.39 × 10^−2^
90SaCo^138^	7657.31–36730.42	75/65	2.93 × 10^−1^	4.00 × 10^−1^
18YoBeHoMa^53^	15702.77–43408.75	9257/9257	8.01 × 10^−4^	2.10 × 10^−2^
91CoSaCo^139^	17898.38–39286.60	328/320	4.79 × 10^−1^	5.50 × 10^−1^
94StBrAb^144^	29998.33–33059.25	562/562	1.42 × 10^−2^	7.00 × 10^−2^
72Engleman^116^	32122.39–35560.02	107/107	1.02 × 10^−1^	3.00 × 10^−1^
93CoChCo^141^	34993.51–46930.92	340/340	1.55 × 10^−2^	2.55 × 10^−1^

For a successful Marvel analysis of transitions data one needs to construct a well-connected SN. It is essential to know the rotational energy levels of the vibrational ground state. Since there are no high-accuracy pure rotational measurements for ^16^OH, we had to rely on calculated energy levels, based on the EH results of 09BeCo.^154^ The calculated lines included are denoted by ‘_C’ in the database.

## 4 Marvel results

### 4.1 ^12^CH

A total of 6348 assigned transitions from 29 distinct data sources were included in our final Marvel analysis of the measured spectra of ^12^CH. From these transitions 5906 lines belong to the principal component, determining 1521 empirical (Marvel) energy levels. Our database contains 49 floating transitions (transitions which could not be linked to the principal component), as well, linking 82 rovibronic energy levels. These floating transitions are retained in the dataset as they might be linked easily to the principal component when new experimental data become available.

After the necessary reassignments, only 393 transitions had to be removed from the dataset considered by the final Marvel analysis, as they are not consistent with the validated transitions. These transitions are retained in the final list of transitions but are given as negative wavenumber entries. Table 4 gives a brief summary of the characteristics of the Marvel results obtained for the seven doublet electronic states studied. The transitions file and the energy levels are given in the ESI.†

**Table 4:** A brief summary of the ^12^CH Marvel results for the different electronic states, the energy and uncertainty ranges are given in cm^−1^. Unc. = uncertainty, Avg. = average

State	*v* range	Levels	Unc. range	Avg. unc.	Range of energy levels
X^2^Π	0–5	711	0.0000–1.3510	0.0252	0.0000–21277.3362
A^2^Δ	0–5	514	0.0051–1.0013	0.1910	23260.1771–39244.0815
B^2^Σ^−^	0–1	109	0.0071–0.5001	0.1026	25712.5053–31474.7352
C^2^Σ^+^	0–2	141	0.0054–0.5001	0.0736	31791.6558–43701.3123
D^2^Π	0–2	58	0.1000–4.1430	0.7270	58999.2647–65867.9341
E^2^Σ^+^	2–2	25	0.5000–2.7250	0.9777	68793.1355–70652.4969
F^2^Σ^+^	0–0	15	0.5000–1.0000	0.7539	64531.9000–64793.2681

### 4.2 ^16^OH

Employing 15 938 measured transitions, we could determine 1624 empirical rovibronic energy levels for ^16^OH. The detailed validation process resulted in the deletion of 119 transitions. The present Marvel database contains 72 floating transitions including 81 rovibronic energy levels. Future high-resolution studies may connect these floating components to the principal one.

Table 5 provides information about four experimentally measured electronic states. It is interesting to note that there are more A ^2^Σ^+^–X ^2^Π than X ^2^Π–X ^2^Π transitions measured (not seen in Table 5). This is solely due to the source 18YoBeHoMa,^53^ in which more than 9200 assigned A ^2^Σ^+^–X ^2^Π lines are given. The transitions file and the energy levels of this study are available in the ESI.†

**Table 5:** A brief summary of the ^16^OH Marvel results for the different electronic states, the energy and uncertainty ranges are given in cm^−1^. Unc. = uncertainty, Avg. = average

State	*v* Range	Levels	Unc. range	Avg. unc.	Range of energy levels
X^2^Π	0–13	1204	0.0000–0.3005	0.0035	0.0000–36721.3447
A^2^Σ	0–9	350	0.0011–0.5000	0.1209	32440.5786–52482.2452
B^2^Σ^+^	0–1	40	0.0051–0.7071	0.0527	68406.2992–69409.1102
C^2^Σ^+^	0–1	30	0.6103–0.7071	0.6845	88261.1865–89690.2761

## 5 Comparison with existing datasets

The Cologne Database for Molecular Spectroscopy (CDMS),^175^ one of the standard spectroscopic databases, contains data neither for CH nor for OH. There are, however, several other spectroscopic datasets assembled for CH and/or OH. Comparisons of these literature datasets with the results of the present study are given next.

The source 96JoLaIwYu^51^ supposedly contains 112 821 calculated lines for ^12^CH, involving the lowest four doublet electronic states. At the time of the writing of this paper, these data are simply unavailable; thus, no comparison could be performed.

### 5.1 MoLLIST^55^ for ^12^CH

**Fig. 2:**
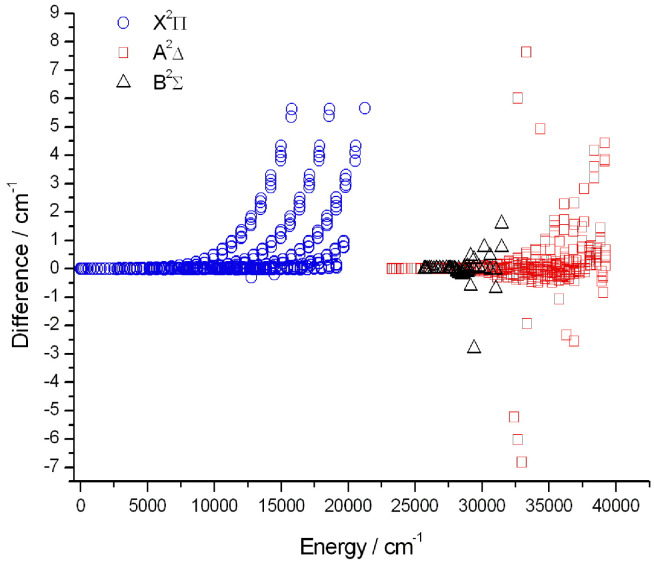
Differences between the Marvel energy levels of this study and the empirical results of 14MaPlVaCo,^9^ concerning the X^2^Π, A^2^Σ^+^, and B^2^Σ^−^ electronic states of the ^12^CH radical.

We used the term values of the CH dataset of 14MaPlVaCo,^9^ downloaded from the VizieR website,^176^ to compare with our final empirical (Marvel) energy levels. Fig. 2 shows the differences between the empirical energy levels of this study and the results of 14MaPlVaCo^9^ for the X ^2^Π, A ^2^Δ, and B ^2^Σ^−^ electronic states.

Unfortunately, we found several conflicts between the positions of newly identified lines, Tables A.2–A.4 of ref. 9, and the official ESI of that study (and the latest version of this database downloaded from the VizieR website^176^). There are conflicts, for example, concerning the labels. To wit, according to Table A.2 of ref. 9, the transition at 22440.19 cm^−1^ is P22ff(17.5), but in the ESI it is designated as P22ee(17.5), *i.e.*, there is a conflict in the rotationless parity. We found several similar cases and we used the labels found in the ESI during this analysis. Furthermore, Table A.3 of ref. 9 contains several non-existent rovibrational energy levels in the B ^2^Σ^−^ state. For example, this table contains P22ff transitions, but in the ^2^Σ^−^ state the parity of an *F* = 2 level is ‘e’.

As seen in Fig. 2, there are a few energies where the deviations are larger than 1 cm^−1^. The reason for these large differences is also the conflict between the 14MaPlVaCo article and its ESI. For example, the difference between the Marvel and the 14MaPlVaCo values for the (A2Delta 3 11.5 e 2) level is more than 6.0 cm^−1^. In Table A.2 of ref. 9, the wavenumber of the P22ee(12.5) line is 22606.47 cm^−1^, but the wavenumber of this line in the ESI is 22612.49 cm^−1^.

### 5.2 MoLLIST^55^ for ^16^OH

**Fig. 3:**
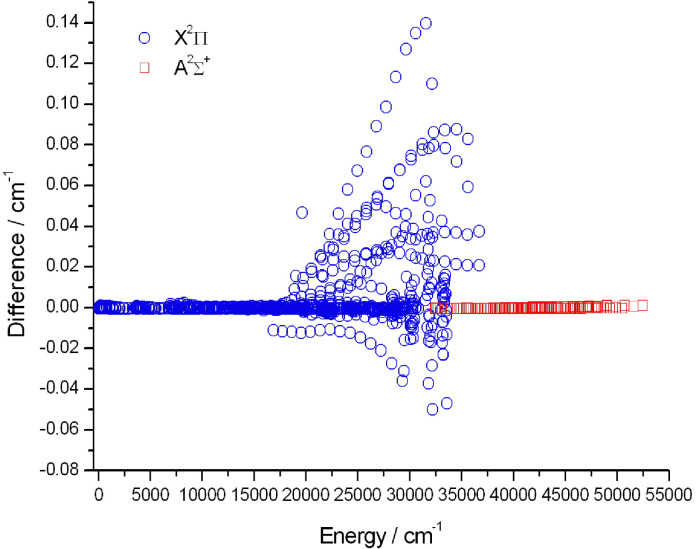
Differences between the Marvel energy levels of this study and the empirical results of MoLLIST,^55^ concerning the X^2^Π and A^2^Σ^+^ electronic states of the ^16^OH radical.

We used the term values of the MoLLIST^55^ OH dataset downloaded from the ExoMol website,^177,178^ to compare with our final empirical energy levels. Fig. 3 shows the deviations between the empirical energy levels of this study and the results of the MoLLIST OH dataset for the X ^2^Π and A ^2^Σ^+^ electronic states.

Fig. 3 shows that between 20 000 and 40 000 cm^−1^ there are several energies where the deviations are larger than 0.01 cm^−1^. It seems that there are conflicts between the measured transitions of 94AbDaRaEn^143^ and 95MeSaGrFa^145^ and the fitted MoLLIST^55^ results.

**Fig. 4:**
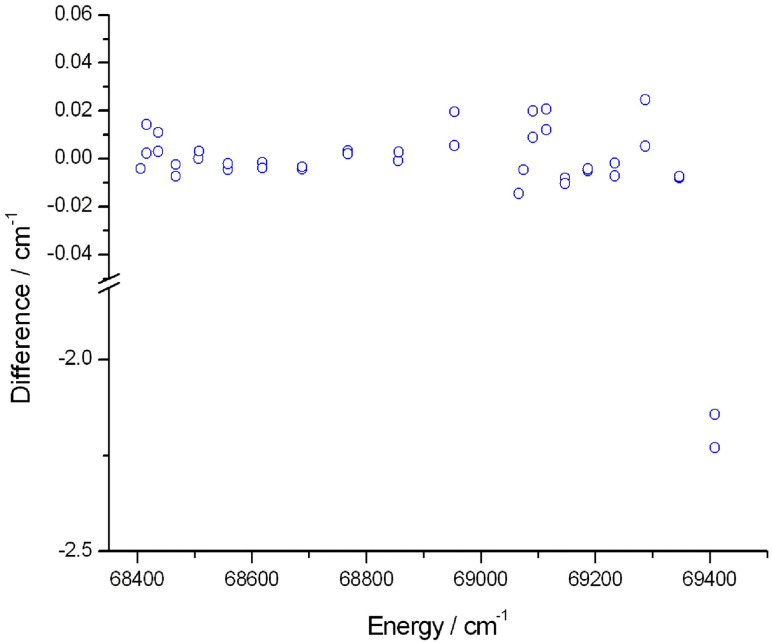
Differences between the Marvel energy levels and the earlier results of 09BeCo^154^ concerning the B^2^Σ^+^ state of the ^16^OH radical.

Furthermore, we compared the term values of 09BeCo^154^ to the Marvel energy levels of the B ^2^Σ^+^ electronic state. Fig. 4 shows the deviations between the Marvel energy levels and the results of 09BeCo^154^ for the B ^2^Σ^+^ electronic state. The Marvel energy levels are determined by the measured transitions of 91CoSaCo^139^ and it is important to note that the authors of 91CoSaCo excluded from the fitting the last two transitions (clear outliers in Fig. 4). This explains why the differences between the Marvel and the 09BeCo^154^ results are much larger in the case of the last two energy levels.

### 5.3 HITRAN and GEISA databases of ^16^OH

The canonical spectroscopic database and information system HITRAN2020^49^ contains 55 698 rovibronic transitions for the ^16^OH radical, involving the X ^2^Π and A ^2^Σ^+^ electronic states and the transitions go all the way up to 43408.75 cm^−1^. Therefore, it can be used to check the completeness of our empirical Marvel dataset. Most of the line positions of the X ^2^Π–X ^2^Π and A ^2^Σ^+^–X ^2^Π bands have been updated in HITRAN2020, using the calculated parameters of 16BrBeWeSn.^52^

Since the line positions of HITRAN2020 are based on effective Hamiltonian calculations, it is not surprising that there are about 43 000 lines that are not available in our Marvel database, which contains almost exclusively experimental information. Of the transitions common to both HITRAN and Marvel, we could reproduce about 13 000 lines within 0.05 cm^−1^. There are 370 HITRAN transitions where the differences between the HITRAN and the Marvel lines are larger than 0.05 cm^−1^.

Similar to the HITRAN2020 database, the recent version of GEISA-2020^50^ is based on EH calculations. This database contains 42 866 rovibronic transitions in the X ^2^Π–X ^2^Π and A ^2^Σ^+^–X ^2^Π band systems up to 35877.03 cm^−1^. The GEISA-2020 database also contains about 25 000 lines that are not part of our experimental database. Since the GEISA database does not contain exact information about the *e*/*f* parity, we could not perform a line-by-line comparison.

## 6 Hyperfine transitions

Although Marvel has been used for the analysis of the spectra of a number of two- to five-atomic species, thus far networks of hyperfine-resolved transitions have not been considered. In those cases where hyperfine-resolved measured lines were available, only their average was utilized.^61,179^ For the radicals ^12^CH and ^16^OH, we decided to retain at least some of the experimental information about the hyperfine lines in the Marvel transitions input file. Most of the published measured transitions are given in MHz; therefore, we keep this unit in our Marvel database.

Note that there are several outstanding papers which report highly accurate spin-rotation splittings within the A and C states of ^12^CH^81,82^ and the A ^2^Σ^+^ state of ^16^OH.^71,72,122,127,130,136^ For example, 86MeUbDy^136^ contains hyperfine-resolved transitions between *e*/*f* doublets in the *v* = 0 vibrational state, 83MeMaMeDy^130^ contains similar data for both the *v* = 0 and *v* = 1 vibrational states of ^16^OH, and 18FaFuMe^157^ provides 12 highly accurate hyperfine-resolved transitions connecting the X and A states of ^16^OH.

None of the hyperfine lines discussed here were employed during the Marvel analysis of the large amount of hyperfine-unresolved data. The issue of how to combine a mixture of hyperfine resolved and unresolved transitions within a Marvel procedure is a subject of active study^180^ and one we plan to address elsewhere.

### 6.1 ^12^CH

To construct the spectroscopic network of the hyperfine-resolved transitions for the ^12^CH radical we collected all available measured transitions belonging to the ground (*v* = 0) vibrational state. The full list of data sources employed in the final hyperfine Marvel analysis of ^12^CH is given in Table 6, which also provides details on the range of frequencies and the number of transitions measured and validated, along with some statistical data about the uncertainties of the sources. This table also shows that we collected 73 transitions from 9 sources. In order to get a complete energy value set up to *J* = 9/2, we had to add 6 calculated lines (published in 01DaEvBr^97^) to the database.

**Table 6:** Experimental sources used to construct the ^12^CH hyperfine spectroscopic network. Given are the frequency range of the validated transitions of each source, the number of actual (*A*) and validated (*V*) transitions, and selected uncertainty statistics. Avg. = average, unc. = uncertainty, and max. = maximum

Source tag	Range/MHz	*A*/*V*	Avg. unc./MHz	Max. unc./MHz
85ZiTu^80^	701.68–724.79	2/2	1.00 × 10^−2^	1.00 × 10^−2^
13TrHeToLe^20^	701.68–3349.19	7/7	9.71 × 10^−6^	2.10 × 10^−5^
73RyElIr^68^	3263.79–3349.19	3/3	3.00 × 10^−3^	3.00 × 10^−3^
06CaMoBrTh^98^	3263.80–14778.96	9/9	2.00 × 10^−3^	3.00 × 10^−3^
83BrBr^74^	4847.84–24482.10	14/14	4.61 × 10^−1^	1.00 × 10^−0^
84BrBr^76^	7274.78–7398.38	4/4	2.63 × 10^−1^	4.50 × 10^−1^
00Amano^96^	532721.33–536795.68	6/6	2.90 × 10^−1^	8.50 × 10^−1^
13TrHeHiTa^102^	532721.59–536795.57	6/6	6.00 × 10^−4^	6.00 × 10^−4^
01DaEvBr^97^	2010810.46–4250352.95	16/15	1.53 × 10^−1^	6.50 × 10^−1^
01DaEvBr_C^97^	3376791.22–4238488.08	6/6	1.37 × 10^−1^	1.60 × 10^−1^

**Fig. 5:**
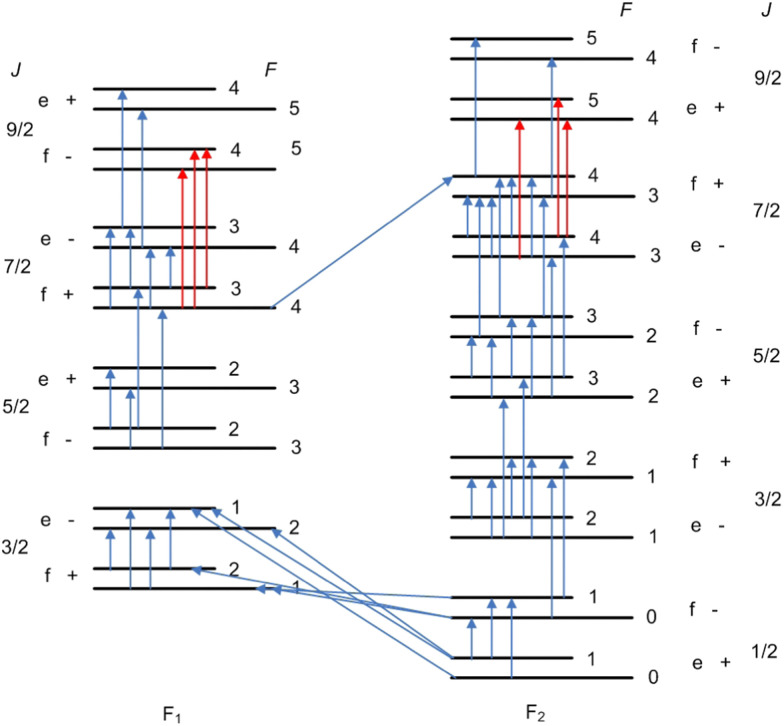
Spectroscopic-network representation of the Λ-doublet and proton hyperfine splittings and the electric-dipole- and magnetic-dipole-allowed transitions measured for the ground electronic (X ^2^Π) and vibrational (*v* = 0) state of the ^12^CH radical. The blue arrows depict the experimentally measured transitions, while the red arrows correspond to calculated ones. See the text for the definition of the labels denoting the states.

Fig. 5 shows the SN of the hyperfine transitions measured for the ^12^CH radical. As also seen there, we cannot reach the *f* and *e* levels of *J* = 9/2 energy states of F_1_ and F_2_, respectively, without the calculated transitions (red lines). Since this Marvel database contains only the hyperfine transitions of the ground (*v* = 0) vibrational and electronic state, the following four descriptors were employed to label the rotational levels: *Ω* (1 for the F_1_ and 2 for the F_2_ component), *J*, the rotationless parity (*e*/*f*), and the total angular momentum *F*. Table 7 contains the first 36 Marvel -determined hyperfine energy levels up to *J* = 9/2. As seen there, there are three hyperfine energy levels within the F_2_ component which are known with an outstanding accuracy of just a few (3–6) Hz.

**Table 7:** Energy values and the corresponding uncertainties of hyperfine-resolved levels of the ^12^CH radical based on transitions data reported in Table 6. Unc. = uncertainty. See the text for the meaning of the *J*, *F*, F_1_, and F_2_ descriptors

*J*	F_1_				F_2_			
	Parity	*F*	Energy/MHz	Unc./MHz	Parity	*F*	Energy/MHz	Unc./MHz
1/2					*e*	0	0.000	0.000
1/2					*e*	1	13.713200	4.24 × 10^−6^
1/2					*f*	0	3277.506647	5.20 × 10^−6^
1/2					*f*	1	3349.192556	3.00 × 10^−6^
3/2	*f*	1	536070.7812	6.00 × 10^−4^	*e*	1	2006762.629	2.00 × 10^−1^
3/2	*f*	2	536073.0819	6.00 × 10^−4^	*e*	2	2006812.83	2.00 × 10^−1^
3/2	*e*	2	536774.7595	6.00 × 10^−4^	*f*	1	2014087.83	2.00 × 10^−1^
3/2	*e*	1	536795.5695	6.00 × 10^−4^	*f*	2	2014161.25	2.00 × 10^−1^
5/2	*f*	3	2193034.61	4.58 × 10^−1^	*e*	2	4592650.00	2.24 × 10^−1^
5/2	*f*	2	2193043.82	4.96 × 10^−1^	*e*	3	4592693.00	2.24 × 10^−1^
5/2	*e*	2	2197913.93	6.38 × 10^−1^	*f*	2	4607406.67	2.24 × 10^−1^
5/2	*e*	3	2197882.44	6.08 × 10^−1^	*f*	3	4607471.96	2.24 × 10^−1^
7/2	*f*	4	4718557.56	4.47 × 10^−1^	*e*	3	7999837.21	2.69 × 10^−1^
7/2	*f*	3	4718571.82	4.86 × 10^−1^	*e*	4	7999876.51	2.45 × 10^−1^
7/2	*e*	4	4729822.75	3.32 × 10^−1^	*f*	3	8024257.39	2.45 × 10^−1^
7/2	*e*	3	4729858.84	5.71 × 10^−1^	*f*	4	8024319.08	2.45 × 10^−1^
9/2	*f*	5	8095348.78	4.66 × 10^−1^	*e*	4	12238325.35	2.93 × 10^−1^
9/2	*f*	4	8095365.91	4.69 × 10^−1^	*e*	5	12238362.39	2.77 × 10^−1^
9/2	*e*	5	8115282.26	3.46 × 10^−1^	*f*	4	12274610.34	2.65 × 10^−1^
9/2	*e*	4	8115321.04	5.80 × 10^−1^	*f*	5	12274669.61	3.32 × 10^−1^

### 6.2 ^16^OH

The SN of hyperfine-resolved transitions of ^16^OH is considerably larger than that of ^12^CH, containing more than 200 experimentally-measured hyperfine transitions, collected from 27 sources. Marvel can only determine the absolute energy of a quantum state if, within the SN, there is a path leading from the given level to the lowest energy level. There are no hyperfine-resolved transitions connecting the different vibrational states of ^16^OH; thus, only the ground (*v* = 0) vibrational state is investigated here.

We used the same four descriptors to label the rotational levels of ^16^OH hyperfine energies as in the case of ^12^CH: *Ω* (
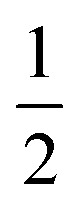
 for the F_2_ and 
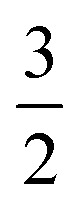
 for the F_1_ component), *J*, the rotationless parity (*e*/*f*), and the total angular momentum *F*. As mentioned earlier, we followed the *e*/*f* scheme and the order of the *F* numbers advocated in 78BrKaKeMi (see Fig. 3 of 78BrKaKeMi^165^). This means that in the ^2^Π_3/2_ component the order of the quantum number *F* inverts above 
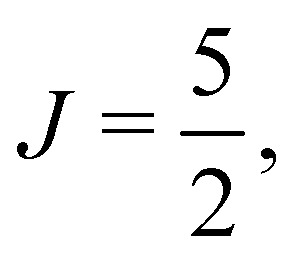
 with larger *F* values belonging to lower energies. For the ^2^Π_1/2_ case the order of (*e*/*f*) parity components swaps for 
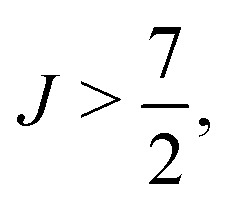
 with the f states lying at lower energy. The full list of data sources employed in the final Marvel analysis of the hyperfine lines of ^16^OH is given in Table 8. This table also provides details on the range of frequencies and the number of transitions measured and validated, along with some statistical data about the uncertainties of the sources.

**Table 8:** Experimental sources used to construct the spectroscopic network of ^16^OH hyperfine lines. Given are the frequency range of the validated transitions of each source, the number of actual (*A*) and validated (*V*) transitions, and selected uncertainty statistics. Avg. = average, unc. = uncertainty, and max. = maximum

Source tag	Range/MHz	*A*/*V*	Avg. unc./MHz	Max. unc./MHz
75DeMa^119^	7.43–23838.93	11/11	2.50 × 10^−2^	5.23 × 10^−2^
75MeDy^120^	88.95–23826.62	17/17	5.88 × 10^−3^	1.00 × 10^−2^
73MeDy^118^	88.95–193.00	4/4	5.00 × 10^−4^	5.00 × 10^−4^
76MeMeMiDy^122^	1171.49–13441.42	11/11	1.82 × 10^−3^	5.00 × 10^−3^
06LeMeHuSa^153^	1612.23–1720.53	2/2	4.75 × 10^−5^	8.50 × 10^−5^
64Radford^109^	1612.23–1720.53	4/4	2.33 × 10^−3^	3.30 × 10^−3^
72MeDy^117^	1612.23–1720.53	4/4	1.25 × 10^−4^	2.00 × 10^−4^
79CoSaAuLe^125^	1612.23–66133.35	12/12	2.55 × 10^−2^	5.00 × 10^−2^
06HuLeSaYe^152^	1665.40–1667.36	2/2	8.00 × 10^−6^	1.20 × 10^−5^
59EhToSt^108^	1665.46–1667.34	2/2	6.50 × 10^−2^	1.00 × 10^−1^
68Goss^111^	1720.53–1720.53	1/1	3.00 × 10^−3^	3.00 × 10^−3^
68Radford^113^	4660.24–6049.08	7/7	6.56 × 10^−3^	1.10 × 10^−2^
70BaDiGoRa^115^	7749.91–7831.96	4/4	5.00 × 10^−3^	5.00 × 10^−3^
55DoSaTo^107^	7760.36–36994.43	12/12	6.31	35.75
77DeMaBaBr^123^	8534.86–70858.93	20/20	3.11 × 10^−2^	1.20 × 10^−1^
80SaVa^128^	13433.96–13442.13	4/4	2.64 × 10^−2^	5.35 × 10^−2^
99ThWuSpMe^148^	13434.00–13442.08	4/4	9.92 × 10^−3^	2.47 × 10^−2^
68PoBe^112^	13434.62–36994.43	8/8	1.15 × 10^−1^	4.78 × 10^−1^
96WuSpMeAn^146^	13434.64–13441.42	2/2	5.00 × 10^−4^	5.00 × 10^−4^
65PoLi^110^	13434.65–13441.41	2/2	2.00 × 10^−2^	2.00 × 10^−2^
53SaScDoTo^106^	23818.16–36994.43	4/4	3.01 × 10^−1^	5.99 × 10^−1^
81KoZoLe^129^	66094.85–70887.99	6/6	3.33 × 10^−2^	5.00 × 10^−2^
93VaEv_C^142^	1834735.02–4602881.87	35/35	7.90 × 10^−2^	1.56 × 10^−1^
13Drouin^156^	1834735.06–2603427.29	17/17	5.34 × 10^−1^	2.37
86BlFaPi^134^	1834735.51–3036645.05	17/17	9.37 × 10^−1^	2.47
86BrZiJeEv^135^	1837816.39–3789214.99	22/22	7.79 × 10^−1^	4.74
93VaEv^142^	1837816.39–4209632.49	13/13	1.20 × 10^−1^	4.00 × 10^−1^
85FaBlPi^132^	2509935.44–2509988.61	3/3	8.04 × 10^−1^	8.08 × 10^−1^

**Fig. 6:**
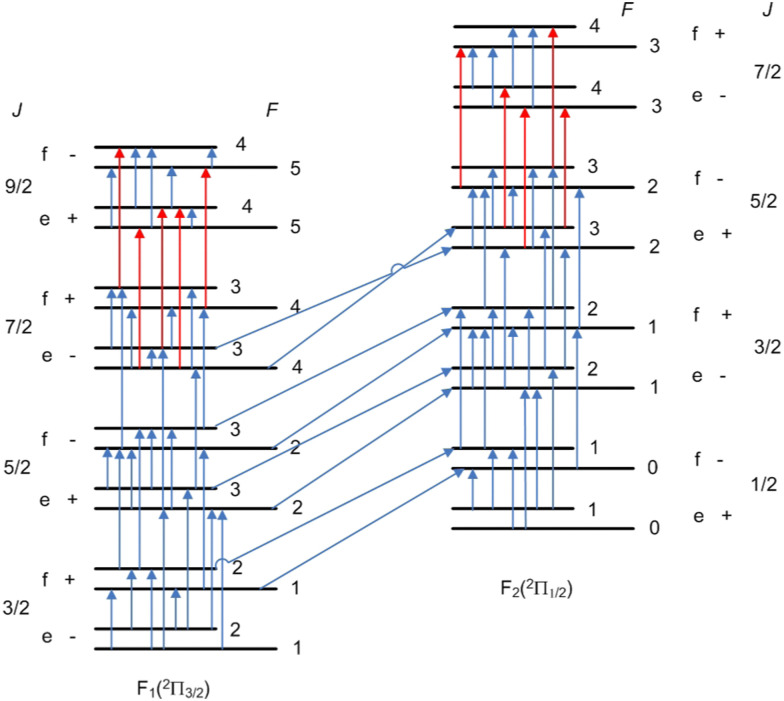
Spectroscopic-network representation of the Λ-doublet and proton hyperfine splittings and the electric-dipole- and magnetic-dipole-allowed transitions measured for the ground electronic (X ^2^Π) and vibrational (*v* = 0) state of the ^16^OH radical. The blue arrows depict the experimentally measured transitions, while the red arrows correspond to calculated ones. See the text for the definition of the labels denoting the states.

Fig. 6 shows the SN representation of the hyperfine transitions of the ^16^OH radical on its ground electronic and vibrational state. As seen there, we can reach the *J* = 9/2 and 7/2 energy levels of the F_2_ and F_1_ components, respectively, if we use the calculated hyperfine transitions of 93VaEv^142^ (red lines).

Table 9 contains the first 32 Marvel-determined hyperfine energy levels. It is interesting to note that the most accurate transition, provided by 06HuLeSaYe,^152^ is 1 667 358 996 ± 4 Hz; therefore, the uncertainty of the (X2Pi 1.5 0 1.5 e 2) level is an outstanding 10^−10^ cm^−1^ (*i.e.*, better than 10^−5^ MHz, see Table 9). There are three hyperfine energy levels in the F_1_ component which have remarkable, about 10 Hz accuracy.

**Table 9:** Energy values and the corresponding uncertainties of hyperfine-resolved levels of the ^16^OH radical based on transitions data reported in Table 8. Unc. = uncertainty. See the text for the meaning of the *J*, *F*, F_1_, and F_2_ descriptors

*J*	F_1_				F_2_			
	Parity	*F*	Energy/MHz	Unc./MHz	Parity	*F*	Energy/MHz	Unc./MHz
1/2					*e*	0	3786170.1	1.56 × 10^−1^
1/2					*e*	1	3786185.0	1.56 × 10^−1^
1/2					*f*	0	3790845.3	1.56 × 10^−1^
1/2					*f*	1	3790935.7	1.56 × 10^−1^
3/2	*e*	1	0.000	0.000	*e*	1	5620920.0	1.56 × 10^−1^
3/2	*e*	2	53.170893	1.08 × 10^−5^	*e*	2	5620931.9	1.56 × 10^−1^
3/2	*f*	1	1665.40180	1.20 × 10^−5^	*f*	1	5628681.8	1.56 × 10^−1^
3/2	*f*	2	1720.52989	1.00 × 10^−5^	*f*	2	5628752.0	1.56 × 10^−1^
5/2	*e*	2	2509987.83	2.84 × 10^−2^	*e*	2	8657190.1	1.57 × 10^−1^
5/2	*e*	3	2510001.83	2.80 × 10^−2^	*e*	3	8657207.9	1.57 × 10^−1^
5/2	*f*	2	2516018.58	2.84 × 10^−2^	*f*	2	8665326.0	1.57 × 10^−1^
5/2	*f*	3	2516036.92	2.80 × 10^−2^	*f*	3	8665397.5	1.57 × 10^−1^
7/2	*e*	4	6053780.92	3.21 × 10^−2^	*e*	3	12869482.9	1.89 × 10^−1^
7/2	*e*	3	6053788.36	3.17 × 10^−2^	*e*	4	12869506.5	1.89 × 10^−1^
7/2	*f*	4	6067222.34	3.21 × 10^−2^	*f*	3	12874956.0	1.89 × 10^−1^
7/2	*f*	3	6067223.00	3.17 × 10^−2^	*f*	4	12875030.0	1.89 × 10^−1^
9/2	*e*	5	10646265.25	8.34 × 10^−2^	*f*	4		
9/2	*e*	4	10646286.57	8.33 × 10^−2^	*f*	5		
9/2	*f*	5	10670091.87	8.34 × 10^−2^	*e*	4		
9/2	*f*	4	10670104.18	8.33 × 10^−2^	*e*	5		

### 6.3 Comparison with the JPL catalog for ^12^CH and ^16^OH

**Fig. 7:**
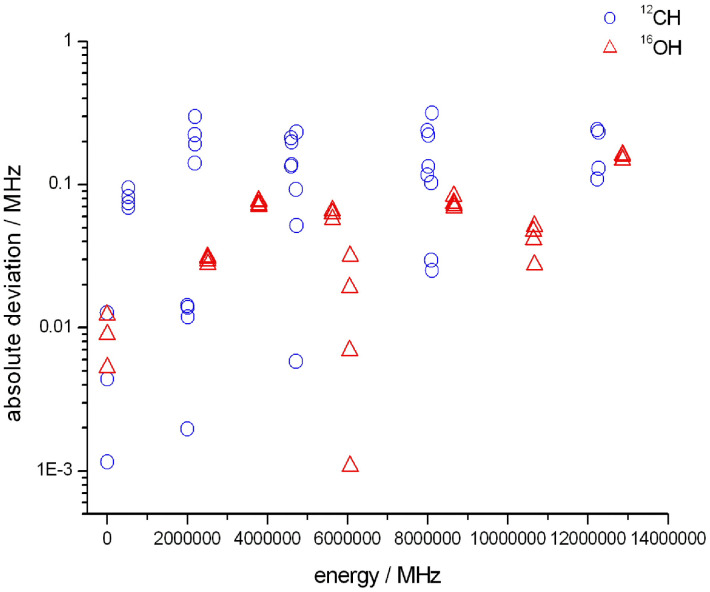
Comparison of the empirical (Marvel) energy levels of this study with those of the JPL dataset for ^12^CH (blue circles) and ^16^OH (red triangles).

The Jet Propulsion Laboratory (JPL) catalog^56^ contains submillimeter, millimeter, and microwave spectral lines in the frequency range between 0 and 10 000 GHz for more than 300 atomic and molecular species. Since the JPL catalog contains both calculated and experimental lines, with the corresponding ‘experimental’ energy levels, it was an obvious choice to use this database to check the quality of the empirical (Marvel) results of this study. Fig. 7 shows the differences between the Marvel and the JPL energy levels, both for ^12^CH and ^16^OH.

For ^12^CH, the JPL catalog lists 58 experimentally measured hyperfine transitions and this dataset was extended with 67 lines of FT-IR measurements,^83^ which are not hyperfine resolved. The accuracy of the lines in the emission spectrum of 87Bernath^83^ is significantly worse than that of the hyperfine measurements; therefore, we decided to consider only the 58 hyperfine lines during the comparison. As Fig. 7 shows (see the empty blue circles), most of the differences between the empirical energy levels of this study and those of the JPL database are less than 200 kHz.

For the OH radical, the JPL catalog lists 3153 experimentally measured transitions, which belong to six isotopologues. From the transitions listed, 739 lines belong to the vibrational ground state of ^16^OH. It is important to note that the JPL database contains results from a far-infrared spectrum (11MaPiBaBr,^43^ with 0.0002 cm^−1^ average uncertainty), an IR spectrum (85LeBoDe,^133^ with 0.001 cm^−1^ average uncertainty), and a solar spectrum (95MeSaGrFa,^145^ with 0.001 cm^−1^ average uncertainty). As seen in Fig. 7, the differences between the Marvel and the JPL energy levels (red triangles) are less than 200 kHz, mutually confirming the data contained.

## 7 Conclusions

Accurate empirical rovibronic energy levels, with dependable, statistically significant, individual uncertainties, are reported for the following seven and four doublet electronic states of ^12^CH and ^16^OH: (X ^2^Π, A ^2^Δ, B ^2^Σ^−^, C ^2^Σ^+^, D ^2^Π, E ^2^Σ^+^, and F ^2^Σ^+^) and (X ^2^Π, A ^2^Σ^+^, B ^2^Σ^+^, and C ^2^Σ^+^), respectively. For ^12^CH, a total of 1521 rovibronic energy levels are determined in the principal component of its measured spectroscopic network (SN), utilizing 6348 experimentally measured and validated transitions. For ^16^OH, after a careful analysis and validation of 15 938 rovibronic transitions, collected from 45 sources, 1624 empirical rovibronic energy levels are determined. Determination of the empirical energy levels is based on the Measured Active Rotational-Vibrational Energy Levels (Marvel) algorithm.

The hyperfine lines measured for the two radicals are included in the Marvel analysis. These accurately measured transitions form floating components within the SN; thus, at the moment, they do not contribute toward improving the overall accuracy of the experimental SNs of ^12^CH and ^16^OH. Nevertheless, in the near future it might become possible to connect the hyperfine-resolved and-unresolved components, see, for example, Bowesman *et al.*^180^ The most accurate line is provided by 06HuLeSaYe^152^ at 1 667 358 996 ± 4 Hz which allows the (X2Pi 1.5 0 1.5 e 2) level to be determined with an uncertainty of only 10^−10^ cm^−1^.

The present database of ^12^CH and ^16^OH transitions and energy levels are compared to several line lists, including the HITRAN2020,^49^ GEISA,^50^ MoLLIST,^9^ and JPL^56^ datasets. This comparison shows an overall satisfactory agreement and also points toward the inaccuracy of a small subset of effective Hamiltonian energies.

The large set of data presented should serve as a starting point to refine the line lists of these radicals. Such attempts have been made by us before, see the case of ^12^C_2_.^181^ We note that the determination of accurate energy levels will allow a large number of new transitions to be predicted with experimental accuracy; in the case of our recent study of formaldehyde (H_2_CO) this gearing led to a more than a twenty-fold increase in the number of predicted transitions relative to the number of unique measured transitions.^182^

## Conflicts of interest

There are no conflicts to declare.

## Supplementary Material

CP-024-D2CP02240K-s001

CP-024-D2CP02240K-s002

CP-024-D2CP02240K-s003

CP-024-D2CP02240K-s004

CP-024-D2CP02240K-s005

CP-024-D2CP02240K-s006

CP-024-D2CP02240K-s007

CP-024-D2CP02240K-s008
